# Extracts and Gleanings

**Published:** 1872-08

**Authors:** 


### PART II.

### Abridged Extracts and Gleanings from our Exchanges

*MULTUM IN PARVO.*

### CHRONIC CHILLS.

During the two last years I have had ample opportunities to test the powers of quinine in curing chronic chills. I have, during this time, experimented a good deal with other remedies, on quite a number of cases, differing widely in temperament, constitution and habits.

I find some who have been afflicted with a malarial trouble for months, and some for years even, that have tried everything prescribed by different physicians, without any permanent benefit whatever, only an occasional release from the paroxysms for a few days. Some of these cases have, through this time, become very anæmic, weak and desponding; while others have enjoyed apparent good health, and been able to perform any kind of manual labor as usual.

Some of these patients had been fed on quinine, in large doses, for two or three months in succession, without any permanent relief. Some idiosyncrate cases have become so affected nervously, by the protracted use of the drug, that they could not bear even a small dose of it; while others could take large doses and scarcely feel it, and still have their chills at the regular turn, as if nothing had been done to prevent them.

The above cases and their treatment prove to my mind that quinine is not a specific (as some would have it) for chills in the chronic form. We *all* acknowledge its *great* virtue in acute cases, and in all cases of periodic fevers, and in many other periodic diseases, of whatever origin; but to say that it *alone* is capable of curing all chronic cases of a malarial origin, is saying too much. It frequently does this work handsomely, when combined with other things: e. g.: I find that the following combination rarely ever fails to cure the purely anæmic cases:

R.—Mur. tr. iron, . . . f oz ss.

Sul. quinine, . . . . dr j.

Aquœ puræ, . . . f oz iijss.—M.

S. Teaspoonful every hour for six hours before the expected chill, on two chill days, or until the paroxysms are broken; then the same dose three or four times a day for 30 days.

If the patients prefer it in pills, I use the following:

R.—Vallett's mas. of iron, . . dr j.
Sul. quinine, . . . . grs xl.
Pulv. opium, . . . . grs iv.—M.
Ft. pil. No. 20.

S. Take one pill every hour for six hours before the chill; after it is broken take one pill 3 or 4 times a day for 30 days or more.

Anæmic cases troubled at the same time with nervous derangement and general debility, weak appetite, and irregular bowels, are admirable improved, and generally cured, by this recipe, which I obtained from the Louisville Dispensary:

R.—Sul. cinchonia, . . . . dr j.
Sul. iron, . . . . dr iv.
Sul. strychnia, . . . . grs ij.
Sul. copper, . . . . grs iv.
Aromat. sul. acid, . . . oz j-ij.
Aquæ distil., . . . oz viij.—M.

S. Teaspoonful 3 or 4 times a day for 30 or 60 days. It sometimes nauseates the stomach if taken before breakfast; this, however, is soon overcome by taking the morning dose an hour after breakfast.

The administration of this preparation should be combined with the cold shower-bath every morning.

The above recipes rarely ever disappoint me, with the anæmic and debilitated, when persevered in, with the help of good diet and proper prudence.

Now comes the other class of cases spoken of, to wit: those whose health is not materially effected by the recurring chills.

And some of these are very obstinate to treat, as there are no particular indications calling for any special treatment. Such cases of course must be very closely scrutinized, in order to ascertain the idiosyncracy, temperament, habits of body, and the peculiar taste of the patients, or the treatment must be guess work.

If I find, in such cases, any strumous tendency, I use iodine or iodide potass, which generally succeeds. If there is any tendency to hepatic or gastric derangement, I use the nitro mur. acid, in six-drop doses every two hours, for 10 or 12 hours before the chill; after that is broken, then take 10 drops three or four times a day, for a month or two.

If I find a gouty or rheumatic diathesis, I give the alkalies, colchicum, &c., or iodide potass., if chronic. If I find any cutaneous troubles, I use arsenic alone.

When I find a case without any of these peculiarities, that won't get well under ordinary treatment, I then guess at opium, chinodine, gelseminum, capsicum, change of diet, habits, vocation, location, &c.

I have cured several cases recently with chinodine, where no debility nor any organic derangements seemed to be present.

I offer these few remarks and recipes to the profession, with the hope that they may benefit some brother who has, like myself, been thwarted and teased by some such obstinate and peculiar cases.

J. D. Tucker, M. D.

---

### A GOOD LOCAL ANÆSTHETIC.

Attentive readers of the Companion will recollect the high recommendation of Dr. Neasley, (October No., 1871), of a combination of rhigolene, oil of peppermint, and colodion, as a specific, which he says, "will, under almost any circumstances, when the part is accessible, relieve the patient *instantaneously; its effects are magical*," in facial neuralgia, hemicrania, odontalgia, and lumbago. On seeing his article, we immediately devised, and have been using ever since, (with unfailing success) the following formula:

| | | |
|---|---|---|
| R.—Albumin of Egg, . . . | | oz j. |
| Rhigolene, . . . . | | dr iv. |
| Oil of peppermint, . . . | | dr ij. |
| Colodion, | | |
| Chloroform, . . . . | aa | dr j.—M. |

Agitate occasionally for 24 hours, and by gelatinization a beautiful semi-solidified opedildoc-looking compound results, which will retain its consistency and hold the ingredients intimately blended for months.

In the above we find a local agent of signal potency in all neuralgic affections, and for the relief of pain generally.

Apply by smart friction with the hand, or gently with a soft brush or mop along the course of the nerve involved.

For irritable ulcers, spread on lint or linen. May also be used by placing a pledget of lint or cotton in a quinine bottle, well saturated with the compound, and applying to the painful part until a smarting sensation is produced.

We mention in connection, we are using equal portions of the above and carbolic acid cerate to a painful open cancer of the face with the happiest results—at least so far as complete control of a ll pain is concerned.

Send around to your druggist and try a bottle.

J. Knox Hodge, M. D.

---

Asthma Cured by Rubbing.—Dr. Geo. Gaskoin says:

In the summer of 1870, I was summoned to a lady suffering from an acute attack of asthma. For several nights she had been restricted to the sitting posture, bent over a table, with the forehead resting on her hands. The distress was very great indeed. She was subject to frequent attacks of the kind, complicated to a very moderate extent with catarrh and bronchitic exudation. Her physician, a gentleman who holds high professional rank, was out of town. Nothing had been omitted in the treatment, which of late was simply palliative. She was recognized as constitutionally asthmatic, and little hope was entertained of permanent amendment. The asthma first occurred on the subsidence of nervous symptoms a few years previous. It had not, as far as I am aware, any marked organic basis. There was observable on the legs an eczematous eruption, under these circumstances, I directed that the chloroform liniment of the British Pharmacopœia should be rubbed briskly into the chest for an hour's space, if possible; and this was done daily by a very efficient attendant, who had sufficient intelligence to comprehend and carry out the treatment. Very early much relief was experienced. On the return of her physician to town at the end of three days, she was already so very much changed for the better that he directed the treatment to be continued. From that time it consisted in the daily repetition of the rubbing process for a month, or nearly so, without aid from medicine, and with little restriction as to diet. Beyond the information I received that she was daily improving, I had really little or nothing to do with her professionally after one or two visits. Under the hands of her attendant, she speedily got rid of the asthma. The patient went out of town in the autumn, and enjoyed perfect health and spirits. She took much walking exercise, with exposure, in the cold of the ensuing winter; and, what is very singular, two years have since elapsed, with no return of asthma.

HOOPING COUGH.—

| | |
|---|---|
| R.—Dilute nitric acid, . . . | dr xii. |
| Comp. tinct. cardamon, . . . | dr iij. |
| Simple syrup, . . . . | oz iijss. |
| Water, . . . . . | oz i. |

S. For a child one or two years old, a teaspoonful may be given ever hour or two.

CROTON-CHLORAL HYDRATE.—The effect of this agent has been tried on the human subject in the Berlin Hospital. In a child to whom it was given, complete anæsthesia of the parts supplied by the trigeminus nerve was produced, while the reflex irritability of the rest of the body was retained. The pulse and respirations were unchanged in number throughout. Further researches on insane persons gave the sams result, and Dr. Liebreich concluded therefrom that croton-chloral-hydrate has the property of inducing profound narcosis without interfering with other organs than the brain; while a correspondingly deep narcosis produced by chloral is accompanied by general anæsthesia and by dangerous lowering of the heart's action.—*Medical Advance.*

[The dose, we learn from practitioners who have used it for insomania, facial neuralgia, and in cerebro-spinal meningitis, is five or six grains for an adult.—*Medical World.*

SUBCUTANEOUS INJECTION OF MORPHIA AND CHLOROFORM.—In El Progreso Medico, April 1, we see it mentioned that Dr. Nussbaum has, from a desire to lessen the dangers of chloroform by diminishing the dose necessary to obtain anæsthesia, made use of subcutaneous injections of morphia simultaneously with chloroform inhalation. The combination of the two substances, it seems, has been studied by Claude Bernard with good results, and has been applied to surgery and also in obstetrics. From experiments made by MM. Labbe and Goujon, of which an account was given to the Academy of Sciences (Gaz. Med. de Paris), it results that, by making an injection of small doses of morphia before giving chloroform, we obtain a far longer sustained anæthesia, with very little chloroform; and it is certain that the dangers of this medicament are in direct relation with the quantity breathed by a patient.—This is a great step, says the Progresso Medico, since great risk is spared to the patient, whilst at the same time the local and general sensibility of the patient is lessened.

VALUABLE TONIC.—

| | |
|---|---|
| R.—Extract conii, . . . . | dr i. |
| Sesqui-oxydi ferri, . . . | dr ij. |
| Tinct, columbæ, . . . | f oz iss. |
| Syr. tolutan, . . . . | f oz ss. |
| Ol. gaultheriæ, . . . | gtt x. |
| Aquæ font., . . . . | f oz ij.—M |

S. Teaspoonful morning and night.

Useful during convalescence and in all debilitated and anæmic cases.

IODINE SALTS IN SYPHILIS.—In a paper on ozœna read before the London Medical Society by Dr. Prosser James, he said that he had obtained important results in syphilitic diseases of the throat, nose and mouth from other iodine salts besides that of potassa. It is clearly not the potash that cures syphilis, and feeling this, he gave full trial to iodide of sodium. Sodium is a constituent of the frame, and is always more easily assimilable than potash. The sodic salt is more pleasant to the taste; weight for weight it contains more iodine than the potassic salt. Iodide of calcicum was also pronounced an excellent preparation. It is easily borne by the system, and much more agreeable to take. It is really desirable that the profession should recognize that all the salts of iodine are not so unpalatable as the one in common use.

—*The Doctor.*

VALUE OF SALT.—This substance is remarkable as constituting the only mineral eaten by man. Not only does it afford an indispensable and wholesome condiment for our tables, but it forms an essential constiuent of the blood, and supplies to the human system the loss sustained by saline secretions. Its antiseptic properties are invaluable; but although it preserves, it ultimately changes and deteriorates the quality of the food to which it is applied, rendering the same innutritious and indigestible; for salt, notwithstanding its being a strong stimulant to the animal fibre, is not convertible into nutriment. This is the cause why sailors who subsist long upon salted provisions are subject to the sea-scurvy. Its medicinal qualities are also remarkable. While all other saline preparations tend to cool, this buc heats the body, and engenders thirst. Some years ago a medical man wrote a *brochure*, in which he condemned the use of salt, attributing to it all the diseases to which flesh is heir. The poor fellow eventually committed suicide. Only lately a book has appeared in which the writer, who is a physician, recommends salt as a sure antidote to the contagion of small-pox. Doctors will of course disagree; but as *Variola* is acknowledged to arise from a diseased or poisoned condition of the blood, the due use of salt may possibly form a safe and effective specific. Salt is not only an agreeable condiment, but also an indispensable requisite. When moderately used it acts a gentle stimulant to the stomach, and gives a piquancy and relish to for food. In Africa the high caste children suck rock-salt as if it were sugar, although the poorer classes of natives cannot so indulge their palates. Hence the expression in vogue among them, "He eats salt with his victuals," signifying that the person alluded to is an opulent man. In those countries where mineral salt is not procurable, and where the inhabitants are far removed from the sea, a kind of saline powder is prepared from certain vegetable products to serve in its stead. Indeed, so highly is salt valued in some places—such as Prester John's country—that from its very scarcity, it is employed as a substitute for money.—*Good Health, February,* 1872, *from Food Journal.*

PILL FOR CONSTIPATION.—

R.—Sanguinariæ pulv.
    Rhei pulv., . . . aa dr j.
    Saponis, . . . . scru ij.—M.

Make 32 pills. S. One pill every morning and night.

Dr. Green remarks: "We are assured by an old and experienced physician, that he has succeeded in overcoming habitual constipation more frequently with this pill than with any other laxative."

TO PREVENT PITTING FROM SMALL-POX.—Dr. A. L. Leach (Medical Times, June 15, 1872), remarks:

I wish to call attention to a preparation that I have used in several cases of confluent variola with great success. It consists in the *carbonate of lead*, the ordinary white-lead of the paint-shops —recommended by Professor Gross, and so successfuly used in the treatment of burns. My mode of using it is to add to the paint a sufficient quantity of linseed oil to make it of the consistence of rich cream, and then, by means of camel hair pencil, apply it to the face. I have done this in the vesicular stage of the eruption, but perhaps it might be well to use it even earlier—in the papular stage. A few applications form a complete covering to the face, at once excluding all possible access of air or light. It is soothing to the parts, allaying irritation, and quieting the inflammation, and in those cases where I have used it has been effectual in preventing the pitting. It commends itself from its easy mode of application, its cheapness, the readiness with which it is obtained, and from the facts that it disposes of the dark room so religiously observed by many practitioners, and that a few applications render all future interference unnecessary.

While upon the subject of small-pox, I will add that for the delirium of this disease, which at times amounts to violence, and is annoying to the friends of the patient and physician, I have found the bromide of calcium of signal benefit.

"JACKSON'S COUGH SYRUP."—According to the Cincinnati Lancet and Observer, the formula for this preparation has not been uniform, and therefore the Cidcinnati College of Pharmacy has recently presented the subjoined one as a uniform standard:

| | |
|---|---|
| R.—Fl. ext. ipecac, . . . | oz ss. |
| Fl. ext. senegæ (oz j. Rad. senegæ to fl oz j) | dr iij. |
| Fl. ext. Rhei, . . . . | dr iv. |
| Syr. simplex, . . . . | oz xxxj. |
| Morphia murias, . . . | gr viij. |
| Ol. sassafras, . . . | gttæ xxxij.—M. |
| Ft. mistura. | |

DR. GAUSTER, OF VIENNA, ON CHLORAL HYDRATE.—In the current number of the Memorabilien, Dr. Gauster mentions a case of tetanic spasms, in a woman pregnant five months, which he succeeded in successfully treating by subcutaneous injection of morphia, and one drachm of chloral given in Liebreich's formula. In seven days of treatment the attack of tetanus were arrested.

Cold Food for Infants.—Dr. J. H. T. King, (Medical Times, June 15th, '72), says:

Instead of giving warm milk, I have adopted the plan of giving cold milk entirely—ordering the babe's bottle to be kept standing in iced water in summer and in cold place in winter. This method I have found, from practical experience, to answer remarkably well. If there is any tendency to diarrhœa, I recommend the milk to be heated to 212° F., and afterwards allowed to get quite cold before being used. In private practice, I am of opinion that bottle-fed infants generally have their food given them too warm.

For Gonorrhœa.—

R.—Cubebs (recently pulverized),
Alcholic extract of cubebs, . aa dr iss.
Make 60 pills.

S. Three pills three times a day, to be increased to four, five or six pills.

This is very efficacious, and generally better borne than any balsamic preparation.

Another:

R.—Powdered camphor, . . . gr vj.
" cubebs,
Alcoholic ext. of cubebs, . aa dr iss.—M.
Make 60 pills.

S. As above.

This is excellent when there is frequent desire to urinate.—*Dr. Henry's Treatment of Venereal Diseases.*

Thoracentesis.—In a recent paper contained in the Bulletin Generale de Therapeutique, vol. lxxx. (The Practitioner for May, 1872), M. C. Paul gives eight cases of acute pleurisy, which, as soon as the diagnosis was ascertained, he at once treated by puncture of the chest. The results were extremely favorable the duration of the disease being materially shortened, all the patients being restored to health in the course of three weeks. M. Paul thinks the mode of treatment especially advantageous for private cases, and gives as a prognostic sign that when the effusion coagulates immediately after being withdrawn there is li[illegible] or no tendency in it to re-collect.

Effects of Opium on Snake-Poison.—A correspondent in The Indian Medical Gazeette for March, 1, 1872, says, "It is well known among Bengalis that opium-eaters can resist, to a certain extent snake-poison."

Hydrarthrosis Treated by Injections of Tincture of Iodine.—Dr. Keppler reports, in the Wiener Medizinische Presse for April 28, a case of hydrarthrosis of the knee-jointtreated by Professor Dittel, of Vienna, by injections containing iodine. The patient was a lad eighteen years of age, who, five and a half years before coming under observation, had received a severe contusion of the knee, which was followed by an accumulation of the synovial fluid of the joint. To relieve this condition, Professor Dittel injected into the joint two scruples of a mixture composed of equal parts of water and of tincture of iodine, which was drawn off ten minutes afterwards. Very few general symptoms followed the operation, although it gave rise to synovitis. Seventeen days later the injection was repeated—this time with pure tincture of iodine, which was allowed to remain. Synovitis was again excited by it, but was this time followed by a decided diminution of the swelling. The operation was again repeated a month later. The patient was discharged three and a half months after the institution of the treatment, completely cured.

For Colic, Flatulence and Spasm in Children.—

| | |
|---|---|
| ℞.—Spiritus Etheris, . . . | gtt 40. |
| " Chloroformi, . . . | gtt 40. |
| Tinct. cardamon comp., . . | fl dr 2. |
| Spiritus myristicæ, . . . | fl dr ½. |
| Olei carni, . . . . | gtt 3. |
| Muc. tragacanthæ, . . . | fl oz ½. |
| Aquæ piperitæ, ad. . . . | fl oz 4.—M. |

S. One or two teaspoonfuls to be taken every 3 or 4 hours, till relief is obtained. For child two years old.—*Tanner.*

Nocturnal Erections.—Dr. Polak describes (Wiener Medizinische Presse, May 19, 1872) a form of nocturnal erection which does not seem to be excited by lascivious dreams, which comes on a short time after the sufferer from it goes to bed, and which is not followed by an emission of semen. In some cases the erection occurs every night, and leaves, next morning, a good deal of sensitiveness of the testicles, so that life becomes a burden in consequence of it. In one case, after the usual remedies had failed, Dr. Polak directed that the patient should, before going to sleep, bandage his penis with a piece of flannel about an inch in width—beginning to bandage at the root of the organ, and when the end was reached returning again to the root. The penis is then to be fastened to the thigh. This was found entirely to prevent the erections from taking place.

HYSTERICA NEURALGIA—COMMONLY CALLED SPINAL IRRITATION.—Dr. J. B. Cory, (Northwestern Medical and Surgical Journal), writes :

In the cases that I have treated, I think I have derived more benefit from blistering the spine, than from any other individual remedy. To relieve pain and quiet nervous excitement, I have been well pleased, in some cases, with hydrate of chloral, given in twenty grain doses, and repeated every two or three hours, if necessary; in other cases, it has not met my expectations. If the tongue is foul, with bad breath, and anorexia, I give small doses of calomel, ipecac and soda, once in three or four hours, until these symptoms are relieved; and whenever the nervous system is sufficiently calmed, and sometimes in the beginning even, I put my patient on a course of nervous stimulants and tonic, with good diet and wine, with occasional blisters to the spine, if it remains tender. The majority of cases, I have found much benefitted by the following:

| | |
|---|---|
| R.—Bromide of potassium, . . | oz ss. |
| Fl. ext. valerian, . . . | oz ij. |
| Spts. ammonia arom, . . . | oz i. |
| Syrup simplex, . . . | oz i. |

S. Take one teaspoonful three or four times a day.

Some case have been benefitted by citrate of iron, quinine, and nux vomica, made into pills, and given three times a day; but on the whole, it has fallen short of my expectations. I am disposed to believe that the syrup of phosphate of iron, quinine, and strychnia, as prepared by Ellis, would be more valuable, though I have not tried it sufficiently to speak intelligently of its merits. The formula of this valuable preparation may be found in Naphey's Modern Therapeutics.

IODOFORM (TERIODIDE OF FORMYL.)—This drug I have administered mostly in combination with iron in anæmic females. Also, in one case of Goitre, its action has been highly satisfactory. The principal diseases for which it has been tried are Phthisis, Amenorrhœa, Syphilis, Glandular Tumors, and Cutaneous Eruptions. In chronic enlargement of Prostrate Gland, M. Moritan used Iodoform as a suppository, one scruple to one ounce of butter, with great benefit to the patient.

Besides the well known effects of Iodine, and its preparations, Iodoform has the advantage of the former preparations, of being stronger, more uniform in its action on the system, and does not act as a local irritant, and can be given uninterruptedly.—*Northwestern Medical and Surgical Journal.*

Cure of Colds.—A Berlin correspondent of the London Druggist' Circular says:

The dreary days of winter have passed away, and as I hope, colds and catarrhs have left likewise, and if I now mention a good cure for these enemies of our winter enjoyments, it may be said that it comes *post festum*, but I believe it is better to be late than not to come at all, inasmuch as the remedy, indeed, has been found very effective, and its application very simple and not unpleasant to the patient. It is prepared in the following manner. A wide-mouth glass-stoppered bottle is filled with amianth, or better, with cotton, and then the following mixture is poured on, so that the cotton or amianth is perfectly saturated with it:

| | |
|---|---|
| R.—Acid. carbolic puriss., 5.0, . . | scru iv. |
| Liq. ammon. caustic, 6.0, . . | dr iss. |
| spec. gravity 0.960. | |
| Aquæ destillat., 10.0, . . | dr ij. scru ij. |
| Spirit. vini. rectificatiss, . . | scru iv. |

The vapors are drawn into the nose frequently during the day, and now and then inhaled into the mouth. A medical gentleman of Stettin, who is renowned not only for his skill as a physician, but likewise for the tremendous catarrh that troubles him every winter, has used this olfactorium anticatarrhoicum with perfect success on his own person, and afterwards on many of his patients, and recommends it highly.

Chinese Treatment of Tetanus.—This mode of treatment of tetanus has been seen by English physicians in China and India to be successful: The patient smokes in a pipe a mixture of from twenty to twenty-five centigrammes of crude opium and tea or rose leaves, which are worked up with a small quantity of molasses. When smoking he must inspire as deeply as possible, and continue this operation until the narcotic influence is noticed. This continues then, as a rule, three or four hours. The smoking is repeated as soon as the tetanic symptoms reappear. In the meantime as much nourishment as possible is given. In using opium thus it must be remembered that its narcotic effect is somewhat neutralized by tobacco.

Bromide of Potassium in Hooping-Cough.—Dr. Beaufort uses the bromide with syr. of Tolu and an alcoholic preparation of aconite, combined together, and by the aid of these three remedies he has seen the disease cured in an average of about twelve days.

Muriate of Ammonia in Bronchitis, Catarrhal Pneumonia, Etc.—In obstinate acute bronchitis, after the first intense stage, in catarrhal pneumonia, both of children and adults, in bronchorrhœa, and also in ordinary chronic bronchitis, the learned editor of New Remedies says he has obtained more apparent good from the muriate of ammonia than any other remedy. Of course, other secondary measures are to be vigorously used—counter irritants, poultices, support or diminution of food supply, &c., as the case may call for. The best formula for giving the muriate is as follows:

R.—Ammonia muriat, . . . dr ij.
Extr. glycerrhiz, . . . dr j.
Mucil. acaciæ, aqua, . . aa f oz iij.

S. Tablespoonful for an adult every two hours—teaspoonful for a child a year old, every three hours.

Sometimes, however, the patient objects to the mixture of sweet and salt, preferring the following:

R.—Ammonia muriat, . . . dr ij.
Aqua, . . . . f oz vi.

Dose as before.

Where the cough is very annoying, 1-20th of a grain of sulphate of morphia, or 10 to 15 minims of tincture of hyosyamus, may be added to each dose.

In bronchorrhœa, the following may at the same time be used by inhalation, twice or thrice daily:

Sat. solution of alum, . . oz vj.
Tincture hyoscyamus, . . oz ss.—M.

*Medical Press and Circular.*

Glycerinized Cotton for Dressing Wounds.—The Journal de Pharmacie states that Professor Gubler, at a recent meeting of the Academy of Medicine, exhibited specimens of wadding prepared by saturating it with a certain quantity of glycerine, which he had found to render it permeable to all medicinal liquids, without causing it to lose any of its suppleness and lightness. He suggested that in this state it might prove a useful substitute for charpie, in the event of a scarcity of that article. Dr. Delaborde has already employed it with advantage. In order to prepare this dressing, it is only necessary to pour a small quanty of glycerine over the square sheet of wadding, and afterwards express it as strongly as possible.

Cyanosis from Nitrate of Silver.—Dr. L. P. Yandell, Jr., (American Practioner, June, 1872), in speaking of two cases treated for syphilis, having cyanosis, says :

Both contracted syphilis, and for tertiary symptoms got iodide of potassium. This drug was given in from ten to sixty-grain doses, thrice daily, for a number of months, in connection with ferruginous or bitter tonics. One of the patients was forced to discontinue the iodide because of its disagreeable effects upon the system. The other took it until all traces of syphilis had passed away, and he increased in flesh under its use. In both cases the fading of the stains was gradual. In the first case there is a faint trace of discoloration remainining, though it is scarcely perceptible. In the second, which was much the darker of the two, there is not a shade of the disfigurement. The iodide of potassium was not given in either case with referenee to the cyanosis, and its beneficial effects were observed by me accidentally more than a year after their occurrence. It may be well to state that both patients were treated by the moist mercurial vapor bath during much of the time that they were using the iodide of potassium, and the abundant diaphoresis may have assisted the action of the iodide. I would suggest therefore for the treatment of nitrate of silver cyanopathia the use of the vapor bath in connection with the iodide of potassium.

Lip-Salve for Excoriations, &c.—

| | |
|---|---|
| R.—Ammoniated mercury, . . | gr vj. |
| Carmine, . . . . | gr i. |
| Soft cerate, . . . . | dr ij. |

Mix exactly.

Used for excoriations, warts, and cracks, superficial ulcerations of the lips, mucus membrane of the cheeks, red parts of margin of nose, and other parts of the skin which are normally red. The parts should be dried before the salve is applied.

Treatment of Blennorrhagia.—Eleven cases of blennorrhagia are related by J. C. Van Wyck, M. D., of Oakland, Cal. (Western Lancet), in which treatment by carbolic acid, in combination with astringents and anodynes, was, he states, as near being a specific in uncomplicated cases as any remedy believed to be such in the domain of the materia medica. So firmly is he convinced that to it alone are to be attributed the gratifying results which have marked every case in which it was employed, that with the next patient who comes under his care he will use the remedy simply diluted with distilled water.

CHLORAL IN THE CURE OF VENEREAL ULCERS.—Soon after the announcement of the employment of chloroform in the treatment of primitive ulcers, either soft or indurated, Dr. Mancesco Accettella instituted a series of experiments which plainly convinced him of the few indications of this preparation in the cure of venereal ulcers. He says: "While busying myself with similar experiments, Liebreich published his observations on hydrate of chloral as actively hypnotic and anæsthetic. From that time I commenced to adopt that preparation as a local remedy, in concentrated solution, upon ulcers of such ancient date that neither the acid nitrate of mercury, nor the carbo-sulphuric paste, nor other efficient caustics had been able to effect a cure. The effects greatly exceed all my expectations. After the first application the deep parts of the ulcer became detached, healthy and normal granulations sprang up, and the ulcer was transformed into a simple sore, with the usual tendency to cicatrization. In sixty-nine cases in which I applied chloral topically, the following results were obtained: seven ulcerated and obstinate abrasions healed in nine to sixteen days; forty-nine soft ulcers in from eight to fourteen days; three soft ulcers, complicated with diptheria, in eighteen to twenty-nine days; five soft ulcers, complicated with phagedæna, in twenty-four to thirty-two days; five primitive infectious ulcers, in fifteen to twenty days. Among the cases of phagedenic ulcers it is necessary to note two, which, for twelve or fifteen months, had resisted all local and general treatment, although the two women affected had the most fresh and florid constitutions. The solution employed was as follows: chloral hydrate, 5 gram. (grs75); aqua, destil, gram. 20 (drs v). This, applied with a pencil to the ulcerated surface, slightly cauterizes, without producing discomfort to the patient. After two or three applications all unhealthy appearance at the bottom of the ulcer disappears, and the sore presents a bright redness which soon produces the most healthy granulations. A considerably diluted solution should be adopted for the cure of abrasions and simple ulcers. Venereal therapeutics may therefore welcome chloral as a topical remedy, *par excellence*, in the gravest form of ulceration, the phagedenic.—*Gazetta Medica Ital. Lomb.*, No. 31, 1871.

TONIC AND ALTERATIVE.—

| | | |
|---|---|---|
| R.—Ferri iodidi, | . . . . | gr 4. |
| Glycerine, | . . . . | fl dr 4. |
| Infusi Calumbæ, ad. | . . . | fl oz 3.—M. |

S. One or two teaspoonfuls three times a day—to a child.

—*Tanner.*

Nitro-Muriatic Acid as an Hepatic Stimulant.—Dr. J. H. Kidder, (Western Lancet, Cal., May, '72) remarks:

Inspector-General Martin, of the British army, thinks that ascites, or at least that form of it dependent upon cirrhosis of the liver, can be entirely and permanently relieved, and the condition of the liver greatly improved, by the use of nitro-muriatic acid, applied systematically. He bases his opinion upon a very extended experience, during a long service in India, the results of which are to be found in the Lancet for April, 1866. The use of nitro-muriatic acid as liver stimulant is, to be sure, no new thing, having often been tried and found wanting in various emergencies; but Inspector-General Martin believes that the fault lies not in the remedy, but in the mode of its application; and he carefully prescribes the method which, in his hands, has proved so successful. I regret that I must quote from imperfect notes, and do so under correction.

The acid should always be made extemporaneously by adding to five parts, by volume, of strong hydro-chloric acid, three parts of nitric. The additions must be made gradually, shaking the bottle well each time. After the acids have been well mixed, the bottle or jar should be left unstopped for twenty-four hours before use. Three (3) fluid-drachms of this acid, diluted with one pint of water form a lotion, which is to be applied over the region of the liver twice a day, with *brisk friction*, while at the same time the feet are immersed in a bath of the same. The hands of the attendants making the application should be protected by oiled silk gloves, else, as stated by Inspector Martin, and verified by my own experience, bilious diarrhœa will result to them, from absorption of the acid through the palms of the hands. If the practitioner please, he may give the acid internally, in the usual doses, but I have found its administration in this manner not effective, and very likely to disturb the already vitiated processes of digestion.

Applied in this manner steadily and perseveringly, Dr. Martin claims that the obstruction to the portal circulation will be so far removed as to prevent entirely the recurrence of ascites, and to secure to the patient a degree of health and comfort not otherwise to be obtained.

Opium vs. Potass. Brom.—Dr. J. M. Da Costa states that the faintness and nausea which frequently follow the administration of opium may be prevented by giving the patient a full dose (grs. 20 to 30) of bromide of potassium about three hours previously. Sound sleep is thus often obtained by patients who would otherwise pass restless nights.—*American Jour. Med. Science.*

Pinus Canadensis.—Dr. W. T. Walker, in the Medical Record, thus speaks of it:

"For the past six months I have used this extract in many cases affecting the mucous membranes, and in almost every case it has given entire satisfaction. In abrasion of the os and cervix uteri, when applied in its full strength by means of a pledget of cotton, I have found it far more satisfactory than tannin or iodine. In endometritis it has proved equally satisfactory. I have used it with perfect success in acute and chronic vaginitis, by applying it in its full strength every other day. I have also used it in several cases of gonorrhœa, and must say that I vastly prefer it to the vaunted remedy of claret and tannin.

"It is certainly a most valuable astringent and tonic; and I doubt not that it will very soon have its place in our Materia Medica."

Combined with a little Tr. Opii. and diluted with water, it has been found highly serviceable in cases of chronic diarrhœa and chronic dysentery, in which it speedily controlled the disease. In fact, in all diseases, where an astringent is indicated, it will be found a superior remedy, especially in gonorrhœa.—*Cincinnati Medical News.*

Cathartic in Albuminuria.—Dr. Moore, of Rochester, N. Y. (Med. and Surg. Reporter), read a paper before the Central New York Medical Society, in which the idea was enforced that albuminuria might remain as an organic disease for a considerable length of time; and during that time there was a remedy in sulphate of magnesia. A case was presented, in which the condition of albuminuria was removed by the cathartic treatment.

Drs. Benidict and Campbell gave histories of cases which had been managed according to Dr. Moore's plan, which afforded relief.

In answer to Dr. Mowris, whether the saline treatment had been successful after scarlet fever, Dr. Moore replied affirmatively.

For Croup and Infantile Pneumonia.—

| | |
|---|---|
| R.—Potassii iodidi, . . . | gr xv. |
| Tinct. assafœtidæ, . . . | fl drs iss. |
| " Senegæ, . . . | fl drs iij. |
| Syrupi mori, ad., . . | fl oz iij.—M. |

S. A teaspoonful every 2, 3, or 4 hours—for a child two years old.—*Tanner.*

Ergot in Paralysis of the Bladder.—*By D. Theodor Roth, Eutin.*—Dr. Roth furnishes the details of a series of cases in which Ergot has proved advantageous in the treatment of this disease. The following is one of the cases he describes: A laborer, aged 49, of powerful build and otherwise in good health, experienced, for sometime previous to seeing Dr. Roth, difficulty in passing water, consisting in frequent desire and pain, whilst on attempting to micturate a delay occurred, which was not overcome or shortened by forcing. When the water at length flowed it only dribbled. On one occasion he had been working for many hours with the feet in wet earth, and on making the attempt, found it impossible to pass any water. Violent efforts to micturate gradually came on, with pain in the lower part of the belly, and he then applied to Dr. Roth. On the introduction of a catheter, a large quanty of urine was evacuated, by which the pain was removed. Twelve doses of ergot were now administered, of about eight grains each, at intervals of three hours. He was told to return if any inconvenience in the passing of the urine was experienced, to keep himself at rest and warm, to take slops, and to evacuate the bladder as soon as he felt any desire. No further treatment, however, was required, and the state of the man, after the lapse of six days, was better than it had been for a long time previously.—*London Practitioner, from Deutsche Klinik.*

Neuralgia of Kidneys and Stomach.—(Dr. C. C. Cox's formula.

R.—Bi-carb. potass. . . . . dr j.
Acidi. hydrocianici, . . . gtt xxiv.
Sol. sulph. morph. . . . gtt xxiv.
Aquœ camphori, . . . . oz iv.—M.
S. Two teaspoonfuls pro re nata.

A New Remedy for Dyspepsia.—Dr. Charles Rice (Am Jour. Pharmacy), says:

Although I call this preparation new, it has been in use for several years in the public hospitals and dispensaries of this city, and also in private practice, and has acquired the reputation of being one of the most prompt and valuable remedies at present known for gastric disturbances, depending upon an abnormal or defective digestion generally, and particularly so for the gastric intolerance of consumptive patients. Its action is often so prompt that one full dose has in many instances afforded immediate relief.

Being requested some years ago to devise a liquid preparation containing bismuth and iron (at that time intended for use in some other complaints), I finally, after various trials, adopted the following formula, which I have followed ever since:

Take of citrate of bismuth, ammonio-citrate of iron, each 320 grs.; water of ammonio, water, each a sufficient quantity.

With 4 oz of water rub the citrate of bismuth into a smooth paste; gradually add water of ammonio until solution has taken place, being very careful not to have an excess of ammonio. Now add the ammonia-citrate of iron and some more water; dissolve, filter, and wash the filter with enough water to make the solution measure 1 pint.

This solution, if intended to be long kept, may be partly made up with glycerin, although I cannot speak from experience whether it is so well borne by the stomach. A more useful addition, however, is good sherry wine, of which there may be used 1 fl. oz. (or perhaps more), in so much water.

The above solution is prescribed under the name of Liquor Ferri et Bismuthi Citratis, and contains in 1 fluid-drachm 2½ grs. each of citrate of bismuth and ammonio-citrate of iron. The dose is from 1 to 2 fluid-drachms, half an hour before meals, or—when required—after meals.

The solution may also be prepared of a concentrated state, and spread upon plates of glass to dry, yielding exceedingly handsome scales of a golden-brown color, which must be protected from the light, and 5 grains of which are equal to 1 fluid-drachm of the solution.

For Torpid Bowels.—

R.—Tinct. sanguinairæ sat.,
Tinct. aloes comp'd, equal parts.
S. 30 to 60 drops twice daily.

It improves the digestive organs and increases peristaltic action.

In Convalescence from Typhus Fever.—Koch is quoted by Dunglison as recommending berberina highly in convalescence from typhus, cholera, &c. Waring refers to the employment of some of the species of berberis in debility after fevers, and in affections of the spleen, especially when given in combination with the sulphate of iron.—*Jour. Materia Medica.*

The Antidote for Phosphorus.—Prof. Bamberger (Wiener Medizinische Press, January 28, 1872), as the result of a series of experiments, has found that oil of turpentine is not an antidote for phosphorous, and believes that in the present state of science the treatment of poisoning by phosphorus by the salts of copper is not only the most rational, but has hitherto been the most successful.

Cerebro-Spinal Meningitis.—Dr. N. S. Davis, in a paper read before the Chicago Medical Society, says, with regard to the treatment of this disease, that he has better success with the Calabar bean, either alone or in combination with ergot, than with any other remedy. For an adult he makes up the two following prescriptions, of which he gives one teaspoonful every two hours, alternating so that the medicine is given every hour: R.—Tinct. Calabar bean, oz jss; fl. ext. ergot, oz ijss; m. R.—Carbolic acid cryst., grs vj; glycerine, oz ss; tinct. gelseminum, oz ss; water, oz iij; m. The medicine to be given without raising the patient's head from the pillow, to avoid the danger of vomiting. Cold cloths are to be kept to the head.—*Med. Exam.*

For Chlorosis, Etc.—

| | | |
|---|---|---|
| R.—Manganessii Iodid, | . . . | dr ij. |
| Tinct. cardamon, | . . . | f oz j. |
| Syr. sarsa., | . . . . | f oz v.—M. |

S. Teaspoonful twice a day.

Or,

| | | |
|---|---|---|
| R.—Manganesii carb., | | |
| Ferri carb., | . . . aa | dr j. |
| Sacch. alb. pulv., | . . . | dr ij.—M. |

Make 15 powders.
S. Two or three each day.
The latter is much used by a distinguished practitioner.

A Speedy Cure for Rheumatism.—Dr. R. H. Boyd states that he cures inflammatory rheumatism in from three to seven days by the following method: He gives first a full emetic dose of ant. et potass. tart., and when this has operated, five drops of tinct. opii and five drops tinct. colchici every three or four hours, and a teaspoonful of a half pint mixture, containing dr. iv. potass. acet. every hour. When the patient becomes very hungry, and is quite free from pain, having fasted several days, he allows two teaspoonfuls of milk or one oyster three times a day, increasing the quantity gradually each day.—*Michigan University Medical Journal.*

Iodide of Starch Poultice for Sloughing Ulcers, Etc.—Take two ounces of starch and mix with six ounces of boiling water, to form a jelly; then add, before cold, half an ounce of tincture of iodine. The poultice is now ready for use.

HOSPITAL GANGRENE AND ITS TREATMENT WITH CAMPHOR-GUM.—A. Netter, in Riemes, is of the opinion that hospital gangrene is a peculiar destruction of the subcutaneous and intermuscular, cellular and fat tissues.

The destroyed mass contains, therefore, much fat. The camphor unites with this at a low temperature, and forms a fluid substance —a camphor oil. This is discharged; and then comes to view the tissue capable of life.

Netter says where there are no anatomical lesions, aponeurosis, fascial and otherwise, neither inflammation, complicated with erysipelas, nor pyæmia, that camphor, early applied and in large quantities, is a sure cure for hospital gangrene. He cites a large number of cases successfully treated in this way. Eight cases that were cured are fully described.

He also tried, with equally good results, camphor powder upon phagedenic chancres that had assumed a gangrenous form.

From his remarks we infer that the spread of hospital gangrene rests upon a ferment, whose life camphor powder seems to cut short.

He tried in two cases the application of quinine and charcoal, but without the desired result.

He considers the disease a purely local one, and that the general *malaise* accompanying it, is only in consequence of local disease, the re-absorption of putrid material. He considers the application of ferr. sesq. chlor. as dangerous, and recommends camphor gum as the most effective remedy in his hands yet tried.

*Medical Examiner.*

DIURETIC.—Useful in anasarca arising from disease of the heart.

| | |
|---|---|
| R.—Vini colchici, . . . | fl dr ij. |
| Tinct. digitalis, . . . | fl dr vj. |
| Potass. iodid., . . . . | dr iiss. |
| Syr. sarsa. comp'd, - . . | fl oz ij. |
| Aquæ puræ, . . . . | f oz iij.—M. |

S. Teaspoonful 3 or 4 times a day.

Or,

| | |
|---|---|
| R.—Elaterii, . . . . | gr v. |
| Digitalis pulv., . . . . | gr xv. |
| Ext. Gentianæ, . . . . . | scr j.—M. |

Make 20 pills.

S. One morning and night.

Granular Lids.—Dr. D. S. Reynolds (American Practitioner, June. '72), says:

In that form of papillary ophthalmia arising from simple catarrhal ophthalmia sulphate of copper, applied once in the course of five or six days, by lightly gliding the stick over the exposed papillæ, followed by the cold, wet compressive bandage, with a collyrium every morning and evening of one scruple of alum to an ounce of water, or one drachm of tannin to the ounce of glycerine, continuing the compressive bandage most of the time during the day, will be fouud successful in most acute cases. If there be manifestations of constitutional disturbance, of course constitutional medication must be promptly instituted.

In chronic papillary ophthalmia, arising from neglected catarrhal conjunctivitis, it will be best to apply muriate of ammonia in substance to the affected membrane at least once a day, and at the same time the collyrium of alum before mentioned. If the disease be a sequel to purulent or "Egyytian" ophthalmia, then there will be observed considerable thickening of the conjunctival membrane from interstitial deposit of plastic matter, the papillæ will not project far beyond the surface, and there will be noticed, upon close observation, a considerable amount of contraction of the orbicularis palpebrarum, constituting permanent blepharospasm, thus greatly endangering the safety of the cornea by friction. In all such cases section of the orbicular muscle, by extending the palpebral fissure outward, and securing the skin and mucous membrane along the whole course of the wound by sutures, will be a prerequisite to the successful treatment. Having performed the tarsorrhaphy or canthoplasty, the only after treatment necessary in most cases will be alum, tannin, or chloride of zinc in solution. The strength of the zinc collyrium should never be greater than three grains to an ounce of water. Generally one grain will be sufficient if applied every two hours during the day.

Another form of papillary ophthalmia is that in which the patients are of strumous taint and much debilitated prior to the attack of conjunctivitis. In this class of cases iodide of iron, quinine, good diet, out-door exercise, and comfortable clothing should be first called into requisition. The local application of turpentine, one half drachm to an ounce of codliver oil, every morning and evening will be about all that is necessary. In fact I believe the very best information for one to possess as to the treatment of papillary ophthalmia and trachoma consists in knowing what not to use or apply to the affected membrane.

CHLORAL IN CERTAIN DISEASES OF THE CHEST.—Dr. Waters (Lancet) finds chloral valuable in bronchitis, especially when complicated with emphysema, for its hypnotic properties. Opium can not be used, for it checks expectoration and increases the liability to death from apnœa. Twenty grains of chloral is the usual dose required for this purpose. This remedy is also useful in allaying cough in chronic or in subacute bronchitis. Give four or five-grain doses in tincture of orange-peel or in syrup of tolu. It is useful again in procuring sleep in cases of asthma, both of the purely spasmodic kind and when complicated with emphysema of the lungs. In phthisis it may be used to procure sleep where morphia or opium in any form is not tolerated.

CHRONIC BRONCHITIS.—(Dr. Horace Green's formula.)

R.—Acidi hydrocyanici medicinalis, . gtt 40.
Morphiæ sulph, . . . gr iij.
Tinct. sanguinariæ,
Vini ipecac, . . . aa f oz ss.
Syr. pruni virginianæ vel mistura amygdal, f oz v.
S. A teaspoonful twice a day.

Dr. Green has found this a most valuable remedy in all pulmonary catarrhal diseases unattended with fever. As the acid is apt to float on the top of the liquid, the phial should be shaken on the administration of each dose.

TRACHOMA.—Dr. D. S. Reynolds (American Pructitioner, June 1872), remarks:

Frequently trachoma and papillary ophthalmia are co-existent. In such cases a collyrium of carbolic acid, three grains to an ounce of water, with perhaps the addition of one grain of sulphate of atropia, instilled into the eye every hour, free and frequent ablutions of warm water, containing one drachm of muriate of ammonia to the quart of water, and a full purgative dose of calomel, will usually produce free muco-purulent discharge, with an abatement of all the symptoms, in a few hours.

TANNIC ACID IN CANCER.—Dr. Wharton, President of the Royal College of Surgeons of Ireland, (Medical Press and Circular, December 27, 1871), recommends tannic acid as a valuable dressing in open cancer. He regards it as possessed of some inherent property by virtue of which it promotes assimilation. It should be liberally applied as a daily dressing.

Opening of Bubo with Caustic Potash.—Dr. McNamara (Indian Medical Gazette—N. Y. Medical Gazette) opens acute bubo by potass fusa instead of the knife, and thus avoids the tedious process of unhealthy granulations. The bubo is first covered with several layers of sticking plaster, in which a hole is made half the size of the intended opening, and the caustic is then rubbed on the exposed skin. The spot is covered with sticking plaster and an opiate administered. A black eschar is formed, which is removed in a few days by a poultice, leaving a healthy ulcer, which soon heals by the ordinary treatment. Dr. McNamara is very confidant that this treatment will always prevent the production of the pale, flabby and unhealthy granulations which are so common in bubo. The patient suffers very little pain from the caustic used in the manner prescribed.

Lotion for Gonorrhœa, Etc.—

℞.—Fresh lime water, . . . oz ss.
Caustic sodæ, . . . . gr i.
Distilled water, . . . oz vj.—M.

Used in local baths, poultices, fomenting lotions, injections, tampons, etc. ; for inflammation of mucous membranes and epidermis, especially in shedding of epithelium (a rag or cloth well saturated, to be applied fresh every hour) ; is excellent in diphtheritic deposits and secretions of mucous pus; in abundant and at the same time quickly fermenting fœted secretions. Of special value when used alternating with carbolic acid, as

℞.—Carbolic acid, . . . dr ss–oz i.
Distilled water, . . . oz vj.—M.

This is excellent in ulcerating sores, and suppurations with fœted smell, as met with in pregnant and parturient women.—*Dr. Henry's Work on Venereal Diseases.*

Ergot in Dysentery.—A French physician claims to have succeeded in the treatment of epidemic dysentery by the use of ergot. He gave 7 or 8 grains every four hours, the cure being effected in ordinary cases in two or three days. After a few doses constipation is produced, which lasts three or four days. Ergotin has the same effect.

Chlorate of Potassa in Chronic Dysentery.—Dr. Shackleton, of St Mary's, Ohio, (Cincinnati Clinic) gives 10 grains chlorate of potass with one-fifth of a grain morphia every four hours in chronic dysentery, he says with great success.

Treatment of Gonorrhea by Warm Water Injections.—Dr. John O'Reilly (American Practitioner), in recommending warm water injections in the treatment of gonorrhœa, says: 1st. That gonorrhœa yields to local treatment, and even water injections. 2d. That water injections or medicated lotions owe their efficiency to their frequent application. 3d. That the common small syringe should be done away with in treating this disease, and none used but those throwing a continuous stream. 4th. That large injections, by fully distending the mucous membrane of the urethra, insure a speedier cure than those less copious.—*Canada Lancet.*

Tympanitis.—Against this formidable complication of typhoid fever, which, unless speedily overcome, is followed by cerebral symptoms from the obstructed circulation, M. Sovet administers an injection of cold water, and when it does not occasion the expulsion of the gas, he follows it with another remedy no less simple—this is a cataplasm upon which table salt is sprinkled. Applied upon the abdomen this occasions intestinal contractions almost instantaneously.

Effects of Bromide of Potassium.—Dr. Julius Levy, of Berlin, writes that if bromide of potassium in drachm doses three times daily, is continued for months, a series of boils will be apt to be produced. He says if some preparation of cinchona be given with the bromide, no boils or other evil sequelæ will arise.—*New York Medical Record.*

Ice.—In chloroformic syncoye, Dr. Baillie recommends the introduction of a piece of ice into the rectum, to re-establish respiratory movements. He affirms that it may be confidently relied upon.—*Indian. Med. Gaz.*

Constipation.—Dr. Henry Field strongly recommends the arsenite of iron in the constipation attending uterine diseases. Oxalate of iron is also recommended.

